# Microarray-based gene expression profiling and DNA copy number variation analysis of temporal fossa arachnoid cysts

**DOI:** 10.1186/1743-8454-7-6

**Published:** 2010-02-26

**Authors:** Mads Aarhus, Christian A Helland, Morten Lund-Johansen, Knut Wester, Per M Knappskog

**Affiliations:** 1 Centre for Medical Genetics and Molecular Medicine, Haukeland University Hospital, NO-5021 Bergen, Norway; 2 Department of Surgical Sciences, University of Bergen, NO-5021 Bergen, Norway; 3 Department of Neurosurgery, Haukeland University Hospital, NO-5021 Bergen, Norway; 4 Department of Clinical Medicine, University of Bergen, NO-5021 Bergen, Norway

## Abstract

**Background:**

Intracranial arachnoid cysts (AC) are membranous sacs filled with CSF-like fluid that are commonly found in the temporal fossa. The majority of ACs are congenital. Typical symptoms are headache, dizziness, and dyscognition. Little is known about genes that contribute to the formation of the cyst membranes.

**Methods:**

In order to identify differences in gene expression between normal arachnoid membrane (AM) and cyst membrane, we have performed a high-resolution mRNA microarray analysis. In addition we have screened DNA from AC samples for chromosomal duplications or deletions using DNA microarray-based copy number variation analysis.

**Results:**

The transcriptome consisting of 33096 gene probes showed a near-complete similarity in expression between AC and AM samples. Only nine genes differed in expression between the two tissues: *ASGR1, DPEP2, SOX9, SHROOM3, A2BP1, ATP10D, TRIML1, NMU *were down regulated, whereas *BEND5 was *up regulated in the AC samples. Three of the AC samples had unreported human DNA copy number variations, all DNA gains.

**Conclusions:**

Extending results of previous anatomical studies, the present study has identified a small subset of differentially expressed genes and DNA alterations in arachnoid cysts compared to normal arachnoid membrane.

## Background

**Figure 1:**
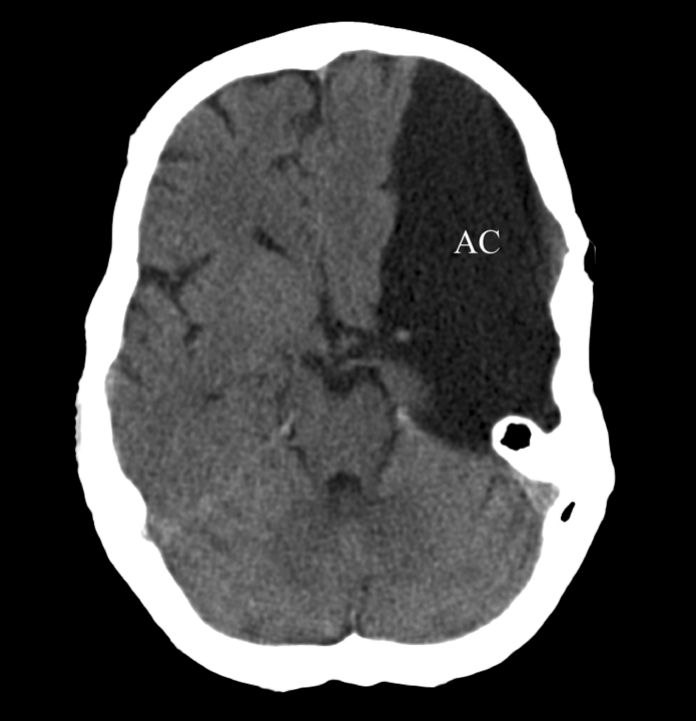
**Radiological presentation of an arachnoid cyst (AC)**. Computed tomography (CT) scan of a large, left-sided temporal AC. Note the splitting of the Sylvian fissure and the compression of the frontal and temporal lobes, all classical features of a Galassi type 3 cyst [10]. The midline is displaced 6 mm to the right. Note the enlargement of the left cranial vault suggesting that the AC was present before the neurocranium was fully developed.

Arachnoid cysts (AC) are relatively common benign lesions, reported to be found in 1.1% of the adult population [1]. Typical symptoms in adults are headache, dizziness, seizures [2] and dyscognition [3]. Although ACs can be found throughout the central nervous system (CNS), they show a marked predilection for the temporal fossa (figure 1) [2,4-6]. In the majority of cases, skull indentations and a large corresponding temporal fossa, as seen on MRI or CT, suggest that ACs arise before the neurocranium is fully developed. Anatomically, ACs are formed by a splitting of the arachnoid mater (AM) [7-9]. The size of ACs is classified according to three Galassi types [10]. Briefly, a type I cyst is small, biconvex, and located at the anterior temporal pole. A type II cyst involves the proximal and intermediate segments of the Sylvian fissure, and a type III cyst involves the entire Sylvian fissure, and has often a marked radiological mass effect.

The cyst wall is composed of non-neoplastic arachnoid cells, and it has been suggested that these cells could secrete cerebrospinal fluid (CSF) [11,12]. The cyst fluid has a chemical composition similar to that of CSF [13-15]. Thus, the frequently seen stable size of ACs might represent a steady state where fluid influx equals the efflux. However, until a proteomic profiling comparing CSF and AC fluid is performed, it remains questionable how similar these fluids are.

Given the likely congenital nature of ACs, it is possible that altered gene expression in neural crest cells at the time of leptomeningeal development may contribute to cyst formation. However, so far there has been very little research on the molecular biology of AC. Hence, putative candidate genes with a role in AC formation are yet to be found. Several methods are available for studying gene expression. As mRNA microarray has the advantage that it can be used to analyze a large number of genes simultaneously, it has evolved to be the method of choice in screening projects of the transcriptome.

Previously, we have characterized the molecular signature of intracranial meningiomas [16]. As these tumors derive from arachnoid cap cells, and normal arachnoid in sufficient quantities is difficult to obtain, we used samples from arachnoid cysts as control tissue. Therefore, it would be interesting to determine whether the gene expression signature of normal arachnoid membrane differs from that of AC membranes. If these signatures were similar, it would imply that AC tissue could be used as a justified source of control tissue in molecular biological studies of conditions involving the arachnoid, such as subarachnoid haemorrhage, meningiomas, and meningitis.

In the current study we have performed a molecular characterization of temporal fossa AC membranes. The aims were: 1) to determine the general gene expression profile of AC samples compared with that of normal AM tissue. In this way we aimed at finding candidate genes with a role in AC development; 2) to search for DNA regions with altered copy number variations (CNV) in AC tissue. The study showed marked similarities in the gene expression profiles of the AC and AM samples; however, we identified a small subset of differentially expressed genes and DNA alterations in arachnoid cysts compared to normal tissue.

## Methods

### Patients and tissue sampling

Tissue from sporadic, non-familial ACs, as well as normal arachnoid, was harvested during surgery and stored in liquid N_2 _in the Bergen neurosurgical tissue bank at the Department of Neurosurgery, Haukeland University Hospital, until analysis. A parallel sample was taken for histological verification of the diagnosis. We obtained written consent from the patients for the study, which was approved by the Regional Ethical Committee. A total of 11 temporal AC were included in the study (table 1), and we used samples from the arachnoid membrane (cisterna magna) of four adult patients as control tissue. These patients were all undergoing posterior fossa surgery for a solitary cerebellar tumour without radiological or intraoperative signs of extension to the cerebellar surface and removal of the arachnoid was part of the surgical procedure. Three of these patients had metastases from adenocarcinoma and one had a hemangioblastoma (not von Hippel-Lindau type). None of the patients had any signs of tumour cell dissemination to the arachnoid, and there was no history or MRI sign of previous haemorrhage in the patient with hemangioblastoma. Because of the very limited amounts of tissue available for analysis, not all the cysts and normal AM were studied with all the techniques; details are given under each method section below (see also table 1).

**Table 1:** Demographic data and techniques used on membrane tissue from 11 arachnoid cyst (AC) patients and 4 arachnoid membrane (AM) patients.

Case #	ID	Gender	Age	Galassi type	Side	Method of investigation
1	AC_2005-056	Male	52	3	Left	mRNA microarray, qRT-PCR, CNV
2	AC_2006-004	Female	34	2	Left	mRNA microarray, qRT-PCR, CNV
3	AC_2006-049	Male	50	3	Right	mRNA microarray, qRT-PCR, CNV
4	AC_2006-051	Female	35	2	Right	mRNA microarray, qRT-PCR, CNV
5	AC_2006-058	Female	33	2	Right	mRNA microarray, qRT-PCR
6	AC_2008-005	Female	27	2	Left	mRNA microarray, qRT-PCR, CNV
7	AC_2008-010	Male	44	2	Left	mRNA microarray, qRT-PCR, CNV
8	AC_39	Male	51	1	Right	qRT-PCR
9	AC_2003_031	Male	54	2	Left	qRT-PCR
10	AC_2004_060	Female	64	3	Left	qRT-PCR
11	AC_2008-004	Male	9	2	Left	qRT-PCR
12	AM_2006-048	Female	82	n.a.	n.a.	mRNA microarray, qRT-PCR
13	AM_2008-008	Male	49	n.a.	n.a.	mRNA microarray, qRT-PCR
14	AM_2006-044	Female	43	n.a.	n.a.	qRT-PCR
15	AM_2006-057	Female	53	n.a.	n.a.	qRT-PCR

qRT-PCR: quantitative reverse transcriptase real-time polymerase chain reaction, CNV: DNA copy number variation analysis, n.a: not applicable.

### RNA extraction and microarray

Seven AC and two controls were studied with mRNA microarray. The details of RNA extraction and microarray analysis have previously been reported [16]. Briefly, we used the Qiagen RNeasy minikit (QIAGEN GmbH, Hilden, Germany) to extract total RNA. After RNA quality and quantity assessment, we then constructed cDNA with RT-PCR reagents (Applied Biosystems, Foster City, USA). Gene expression microarray analysis was performed on the ABI 1700 Expression Array System (Applied Biosystems) using the Applied Biosystems Chemo luminescent RT-IVT Labeling Kit and Human Genome Survey Microarray V1.0.

### Analysis of microarray data

Signal intensities generated with the ABI 1700 Expression Array System were imported into the J-Express Pro 2.7 software (MolMine AS, Bergen, Norway) [17], where inter-array quantile normalization was performed to minimize the effect of external variables on the data. All control spots and flagged spots were removed, leaving 33096 gene probes available for analysis. First, we performed an unsupervised hierarchical cluster analysis in which the group belonging to the samples was defined. Second, we used Significance Analysis of Microarrays (SAM) with 400 and 1000 permutations to compare AC and AM samples [18] and generate gene lists of differentially expressed genes between these groups. With SAM, the false discovery rate (FDR) of the gene lists was calculated. FDR returned the number of false positive genes present on the gene list. A measure of FDR is the Q value, which conveniently shows an estimation of the FDR in percent. In the current study only genes with a Q value <1.0% were accepted as being differentially expressed. The microarray files are deposited at the NCBI Gene Ontology Omnibus (http://www.ncbi.nlm.nih.gov/geo; GEO accession # GSE19727).

### Quantitative reverse transcriptase real-time polymerase chain reaction (qRT-PCR)

Eleven AC and four AM samples were analyzed with qRT-PCR [16,19]. Briefly, gene specific primers and MGB-probes were obtained from Applied Biosystems AssayOnDemand (AOD). Quantification of specific mRNA was performed using the ABI 7900 instrument (Applied Biosystems). β-actin probes were used for endogenous normalization control to adjust for unequal amounts of RNA. Statistical comparisons were performed using the Mann-Whitney rank sum test with the GraphPad Prism™ v.4.0 software (GraphPad Software, Inc. La Jolla, USA). *P*-values were two-sided and considered significant when <0.05.

### DNA purification and copy number variation (CNV) analysis

Six AC samples (AC5056, AC6004, AC6049, AC6051, AC8005, and AC8010) were included in the CNV analysis, which was performed using the Affymetrix Genome-Wide Human SNP array 6.0 (Affymetrix, Santa Clara, USA). DNA used in the CNV experiment was purified from the tissue used for RNA purification and 350 μl of the flow through fraction from the RNEasy column was mixed with 1 μl acrylamide (5 ng/μl) and 500 μl ethanol. The precipitated DNA was collected by centrifugation at 14000 *g *for 10 min. The precipitated DNA was dissolved in 2 ml G2 buffer (Qiagen) and purified using the Qiagen DNA mini kit (cat. no. 51304). Next, 250 ng dsDNA was restricted in each of two separate reactions with NspI and StyI. NspI and StyI adaptors were then ligated to the DNA fragments with T4 DNA ligase before PCR. After PCR, 3 μl of both NspI and StyI products were run on a 2% agarose gel. A smear between 200 and 1100bp was found. The PCR products were cleaned and the DNA concentration measured with NanoDrop (Thermo Scientific, USA). Thereafter, purified PCR products were fragmented and hybridized to the Affymetrix 6.0 chip. The chips were scanned and data processed accordingly with the Genotyping Console. CNVs were crosschecked with The Database of Genomic Variation (http://projects.tcag.ca/variation/[20]).

## Results

### RNA Microarray analysis

The unsupervised hierarchical cluster analysis did not detect any subgroups of the AC samples based on the general gene expression. With SAM the gene lists derived with 400 and 1000 permutations returned identical results. The top 100 genes calculated with SAM had a Q value of 10.4 (see additional file 1: Top 100 candidate genes derived from Significance Analysis of Microarrays (SAM) comparing arachnoid cysts (AC) and arachnoid membrane samples). We found nine genes matching a Q value <1 (table 2). All these genes had fold change differences ≥ +/- 2.6. As seen by the average expression signals, all these genes had signal intensities either for AC, AM, or both above the anticipated background noise (>1000).

**Table 2:** Differentially expressed genes separating arachnoid cysts (AC) from normal arachnoid membranes (AM) determined by microarray analysis.

ABI 1700 Probe ID	Gene symbol	Gene name	Average signal AC	Average signal AM	Fold change	Q value
220229	ASGR1	asialoglycoprotein receptor 1	233	2009	-8.6	0
166918	DPEP2	dipeptidase 2	378	2571	-6.8	0
201106	SOX9	SRY (sex determining region Y)-box 9	871	9030	-10.4	0
207317	SHROOM3	shroom family member 3	222	3188	-14.4	0
167968	A2BP1	ataxin 2-binding protein 1	220	1197	-5.4	0
137724	ATP10D	ATPase, Class V, type 10D	30740	88307	-2.9	0
158289	TRIML1	Tripartite motif family-like 1	296	1040	-3.5	0
130750	BEND5	BEN domain containing 5	10888	4270	2.6	0
102108	NMU	neuromedin U	228	1537	-6.7	0

Key: Average signal intensities for the AC and AM groups are shown in column 4 and 5. Differentially expressed genes were identified by Significance Analysis of Microarrays (SAM) using 400 permutations. The Q value (%) represents the number of false positive candidate genes in the gene list derived from SAM.

### Quantitative RT-PCR

**Figure 2:**
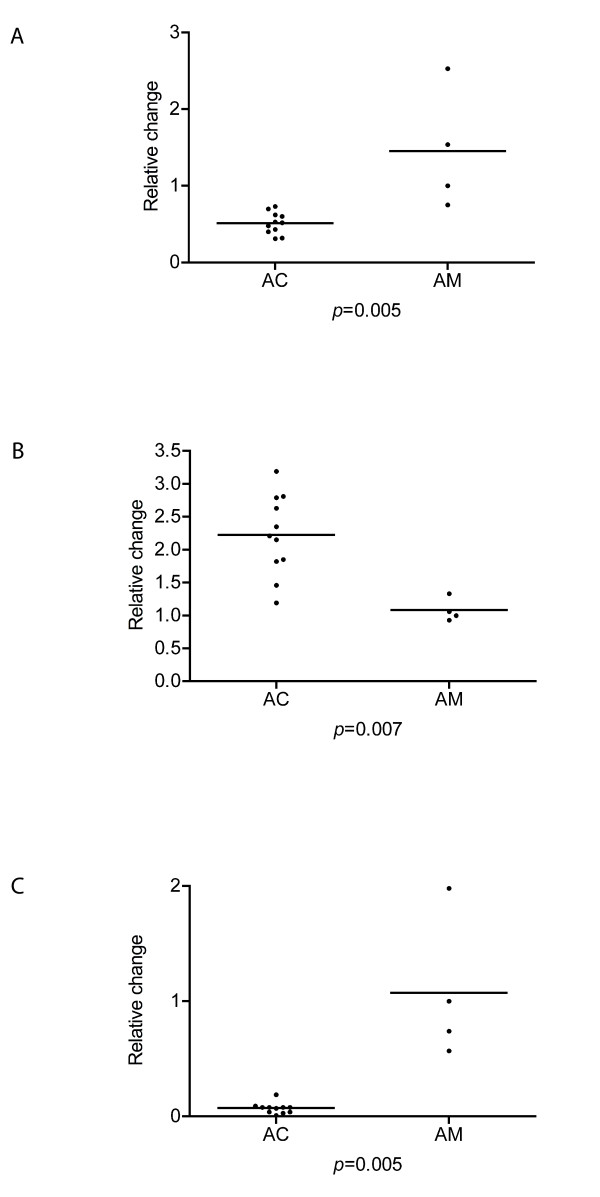
**Validation of differentially expressed genes by qRT-PCR**. Plots showing the expression of three genes analyzed by quantitative reverse transcriptase real-time polymerase chain reaction (qRT-PCR) for arachnoid cyst tissue (AC, n = 11) and control arachnoid tissue (AM, n = 4) for three genes: A) ATP10D, B) BEND5, and C) SHROOM3. The expression of all three genes was significantly different between the two tissues.

**Figure 3:**
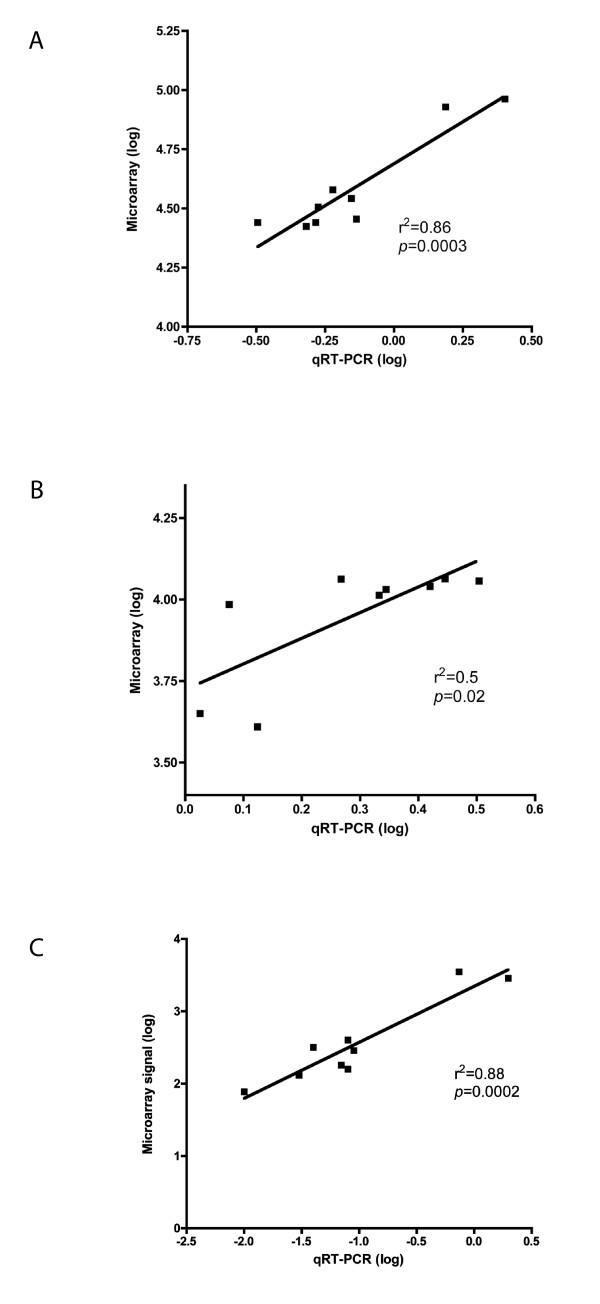
**Correlation analysis between microarray signal and quantitative reverse transcriptase real-time polymerase chain reaction (qRT-PCR)**. Plots showing the correlation between the microarray signal and the expression profile from qRT-PCR analyses for three differentially expressed genes separating arachnoid cysts (AC, n = 7) and arachnoid membranes (AM, n = 2): A) ATP10D, B) BEND5, C) SHROOM3. Data were log transformed before the Pearson correlation was calculated.

The following genes differentially expressed in the SAM analysis were also analyzed with qRT-PCR to validate the microarray results: *ATP10D, BEND5, SHROOM3, SOX9*. For all the studied genes except SOX9 (*p *= 0.09), the difference in expression profile between AC and arachnoid membranes was statistically significant (*ATP10D: p *= 0.005, *BEND5: p *= 0.007, *SHROOM3: p *= 0.005, figure 2). We also investigated the correlation between qRT-PCR and microarray analyses for the expression of these three genes, and found a significant correlation for all three (r^2 ^= 0.8, *p *= 0.0003; r^2 ^= 0.5, *p *= 0.02; r^2 ^= 0.8, *p *= 0.0002, respectively, figure 3).

### DNA copy number variation (CNV) analysis

In DNA purified from AC samples we found several CNVs already registered in the Database of Genomic Variants. However, for three of the samples (AC_2006_051, AC_2005_056, AC_2006_004) we found 14 CNVs with no current reports in the database (table 3). All CNVs in our samples were chromosomal gains. The unreported CNVs were also cross checked against a population of 361 Norwegians with no history of AC, whose genome had been examined for the presence of CNVs with the same platform used in our experiment. None of the 14 AC CNVs were found in these control samples.

**Table 3:** Three arachnoid cyst (AC) samples with chromosomal gains detected with DNA copy number variation (CNV) analysis.

Sample	CNV present in sample	**CNV in region registered in the Database of Genomic Variants**^a^	Genes known to be present in region
AC_2006-051	arr 7p22.1(6498207-6600621)x3 arr 8p11.21(42175206-42281148)x3 arr 14q23.3(64978487-65093419)x3	Inversion and deletion No reports Inversion	GRID2IP, C7orf26, ZDHHC4 IKBVB, PLAT FUT8

AC_2005-056	arr 12q13.13(51746148-51886072)x3 arr 12q24.31(121446657-121547275)x3 arr 14q23.3(64978487-65105576)x3 arr 15q15.3(42468491-42598658)x3 arr 16p13.3(3431656-3522375)x3	Small gains, inversions, and loss spanning region No reports Inversion No reports Loss	ITGB7, ZNF740, CSAD, SOAT2, IGFBP6, SPRYD3 ZCCHC8, CLIP1 FUT8 CTDSPL2, CASC4 CLUAP1, C16orf90, NAT15, ZNF597

AC_2006-004	arr 10p12.31(21822848-21949193)x3 arr 11q12.3(62557724-62669857)x3 arr 12q13.3(51724073-51844567)x3 arr 16p13.3(3522375-3631656)x3 arr 17q21.31(38560627-38814457)x3 arr 19p13.12(14246734-14391382)x3	Loss Loss Loss Loss Loss Loss	MLLT10, C10orf140, C10orf114 SLC22A24 SOAT2, IGFBP6, CSAD, SPRYD3, TENC1 BTBD12, NLRC3, CLUAP1 NBR1, LOC100130581, TMEM106A, DDX39, CD97,

^a^Information extracted from the Database of Genomic Variants (http://projects.tcag.ca/variation/?source=hg18) as of October, 2009 [20].

## Discussion

We studied the global gene expression signature of temporal fossa AC and normal arachnoid membranes with the aim of detecting differently expressed genes. The main finding, however, was a near-complete similarity, by unsupervised cluster analysis and SAM of 33096 individual gene probes. Nevertheless, using SAM we found nine genes with differential expression between AC and AM samples. These nine genes (*ASGR1, DPEP2, SOX9, SHROOM3, A2BP1, ATP10D, TRIML1, BEND5, NMU*) are novel candidate genes that might be associated with the pathogenesis of temporal fossa ACs.

In mice it has been shown that mutations in the SHROOM3 gene result in exencephaly, acrania, facial clefting, and spina bifida; all presumably due to failed closure of the neural tube [21]. The authors suggested that *SHROOM3 *is involved in the regulation of cytoarchitecture that is required for proper neurulation. Another study, has found expression of *SHROOM3 *in several human tissues including the brain [22]. The uniform down-regulation of SHROOM3 in the AC samples is an interesting finding that indicates that specific inactivation of *SHROOM3 *in foci of arachnoid cells may contribute to the development of cysts.

*SOX9 *is a transcription factor located at chromosome 17q24, and inactivation of one allele causes campomelic dysplasia (CD) [23]. CD patients typically have short, bowed long bones, craniofacial defects and female to male sex reversal. Interestingly, cystic hygroma and cystic kidneys have been reported in CD [24]. In the developing brain it is expressed in neural crest cells; the very same cells that differentiate into meninges, and animal studies have shown that inactivation of *SOX9 *in these cells results in craniofacial maldevelopment [25].

From the literature, none of the remaining differentially expressed genes are reported to have known functions linking them to cystogenesis or neural tube development. However, this does not imply that the genes have no function in AC formation as the biological function of many genes are poorly characterized and many genes may have different or multiple functions in different tissues at different stages during the life cycle. Therefore, more research is needed to explore the properties of these genes in humans. Hence, based on current but incomplete knowledge, the most promising candidate genes with a role in the formation of ACs are *SHROOM3 *and *SOX9*.

For validation of the differentially expressed genes, we selected four of them for qRT-PCR: *ATP10D*, *BEND5*, *SHROOM3 *which were significantly altered in the ACs (*p *= 0.005, *p *= 0.008, *p *= 0.005, respectively), and *SOX9 *which showed the same trend, but did not reach significance (*p *= 0.08). The expression of *ATP10D*, *BEND5*, *SHROOM3 *in the qRT-PCR study correlated significantly with that of the microarray study; i.e. a low expression found in the AC by microarray was similarly low by qRT-PCR. This adds internal validity to our microarray platform and thus to the results in general.

Microarray-based CNV analysis was used to study chromosomal aberrations in a detailed manner; with the platform used in our study, more than 946,000 copy number probes span the genome. Thus, submicroscopic DNA regions with duplications or deletions can be identified. Such regions might contain genes of importance for the formation of the cysts. Because of limited amounts of tissue only six AC samples were subjected to CNV analysis. In three of these cases we found a total of 14 CNVs so far not reported in the human genome: all of which were chromosomal gains (table 3). In our samples, the CNVs were located on chromosomes 7, 8, 10, 11, 12, 14, 15, 16, 17, and 19, and several genes are known to be located in these regions. The CNV results were matched against the publicly available Toronto database, as well as against a population based Norwegian sample of 361 individuals located in our laboratory. Although there were no mutual CNVs present in all the AC samples, this does not weaken their potential role. On the contrary, a growing body of evidence from studies on schizophrenia and autism suggests that different DNA alterations on different chromosomes may ultimately contribute to the development of the same phenotype [26-28]. This illustrates the complexity of the genome and its regulation in health and disease, and suggests that similar mechanisms may underlie the pathogenesis of AC.

Gene expression research on diseases affecting the arachnoid membrane (e.g. meningiomas, post-SAH inflammation and meningitis) requires normal arachnoid tissue as control. Since normal arachnoid is more difficult to obtain than AC tissue in quantities sufficient for gene expression analysis, one could ask whether ACs could be used in this research as a substitute for normal arachnoid membrane. In the current study we have shown that the vast majority of genes had similar expression profiles in AC and normal arachnoid membranes. If adjusted for the nine differentially expressed genes, our results suggest that AC tissue may be used as control tissue in such studies.

A limitation of the present study is the low number of normal AM samples used as control tissue. At time of study we had only two AM samples sufficient for cDNA microarray and four AM samples for qRT-PCR analysis. Because of this we had to use strict criteria in the data analysis of the microarray results for the detection of differentially expressed genes. However, when we validated the genes with qRT-PCR and included additional AC and AM samples, the microarray findings were confirmed. As we also have shown, we found a significant correlation between the qRT-PCR and microarray results. Furthermore, all AC samples were supratentorial and the AM controls infratentorial. Thus, one might argue that the differential gene expression may rely on purely anatomical grounds. However, it is our opinion that structurally there is no difference between supra- and infratentorial arachnoid, and that if this were true many more genes would be differentially expressed.

## Conclusions

In summary, this is the first study on the mRNA gene expression in intracranial AC membranes and we have shown that for the vast majority of genes, the expression profiles in AC and AM samples were similar. However, our study showed that a small subset of genes was differentially expressed in the AC samples compared to normal tissue. The differential expression level of these genes might be important for the development of ACs. We also found duplicated DNA regions. Particularly, the functional role of the altered gene expression for *SHROOM3 *and *SOX9 *must be further studied in model systems and the findings of duplicated chromosomal regions have to be verified in replication studies containing more samples.

## Competing interests

The authors declare that they have no competing interests.

## Authors' contributions

MA performed the data analysis and drafted the manuscript. CAH and KW provided surgical specimens and revised the manuscript. ML-J participated in the design of the study, interpretation of data, and drafting of the manuscript. PMK conceived the study, participated in the design and data interpretation, and drafting of the manuscript. All authors have read and approved the final version of the manuscript.

## Supplementary Material

Additional file 1 **Top 100 candidate genes derived from Significance Analysis of Microarrays (SAM) comparing arachnoid cysts (AC) and arachnoid membrane samples**. The rows represent the top 100 genes derived from Significance Analysis of Microarrays (SAM). In the columns the signal intensities generated from the microarray analysis are provided for each of the samples (arachnoid membranes, AM, n = 2; arachnoid cysts, AC, n = 7). Gene identifiers, cytoband, and biological processes according to the PANTHER database (http://www.pantherdb.org) are provided for each gene. Where no information on gene function or name is currently known, the term "null" is used. Click here for file
