# Supplementary material for: Microarray-based gene expression profiling and DNA copy number variation analysis of temporal fossa arachnoid cysts
Source: Cerebrospinal Fluid Res. 2010 Feb 26;7:6. doi: 10.1186/1743-8454-7-6 (PMC2841093; doi:10.1186/1743-8454-7-6)
Supplement: Additional file 1 — Top 100 candidate genes derived from Significance Analysis of Microarrays (SAM) comparing arachnoid cysts (AC) and arachnoid membrane samples. The rows represent the top 100 genes derived from Significance Analysis of Microarrays (SAM). In the columns the signal intensities generated from the microarray analysis are provided for each of the samples (arachnoid membranes, AM, n = 2; arachnoid cysts, AC, n = 7). Gene identifiers, cytoband, and biological processes according to the PANTHER database (http://www.pantherdb.org) are provided for each gene. Where no information on gene function or name is currently known, the term "null" is used. [file 1743-8454-7-6-S1.PDF]

Additional file 1. Top 100 candidate genes derived from Significance Analysis of Microarrays (SAM) comparing arachnoid cysts (AC, n=7) and arachnoid membrane samples (AM, n=2).

#	GENE_ID	Gene_Symbol	Gene_Name	Cytoband	Panther_Process	AM_2006-048	AM_2008-008	AC_2005-056	AC_2006-004	AC_2006-049	AC_2006-051	AC_2006-058	AC_2008-005	AC_2008-010
1	hCG1985284	ASGR1	asialoglycoprotein receptor 1	17p13.2	Cell adhesion-mediated signaling;Cell adhesion;Intracellular protein traffic|Signal transduction|Blood clotting|Receptor mediated endocytosis;Immunity and defense|Endocytosis|Macrophage-mediated immunity;Immunity and defense|Cell communication	1967	2052	148	419	141	212	191	205	320
2	hCG1811727.2	DPEP2	dipeptidase 2	16q22.1	Proteolysis;Other metabolism|Protein metabolism and modification	2580	2563	589	396	237	438	250	518	222
3	hCG27310.2	SOX9	SRY (sex determining region Y)-box 9 (campomelic dysplasia, autosomal sex-reversal)	17q24.3-q25.1	Nucleoside, nucleotide and nucleic acid metabolism|mRNA transcription regulation|mRNA transcription	9564	8496	1242	330	888	1847	589	489	718
4	hCG2027596	SHROOM3	null	4q21.1	Other intracellular signaling cascade;Signal transduction|Cell communication;Developmental processes|Intracellular signaling cascade|Signal transduction	2862	3514	77	159	130	401	181	319	287
5	hCG1811870.1	A2BP1	null	16p13.3	Developmental processes	1227	1167	282	190	315	193	118	240	204
6	hCG2027083.1	ATP10D	ATPase, Class V, type 10D	4p12	Cation transport|Lipid and fatty acid transport;Transport|Lipid, fatty acid and steroid metabolism|Ion transport	91746	84869	27597	34863	37916	26562	27612	32102	28532
7	hCG2039341.1	TRIML1	null	4q35.2	Proteolysis|Protein metabolism and modification	1043	1037	360	214	406	275	260	307	253
8	hCG38197.3	BEND5	chromosome 1 open reading frame 165	1p33	null	4071	4469	9656	11407	10742	10975	11554	10315	11574
9	hCG20599.3	NMU	neuromedin U	4q12	null	1720	1356	189	328	180	174	138	232	355
10	hCG39524.3	BMP2	bone morphogenetic protein 2	20p12	Skeletal development|Mesoderm development|Developmental processes	3049	2658	849	161	388	437	328	818	484
11	hCG37191.2	GATA6	GATA binding protein 6	18q11.1-q11.2	Nucleoside, nucleotide and nucleic acid metabolism|mRNA transcription regulation;Developmental processes|Endoderm development|mRNA transcription	9687	13582	2156	1691	1246	1575	592	1513	411
12	hCG1818780.1	ANTXR2	anthrax toxin receptor 2	4q21.21	Immunity and defense	19534	22162	8419	8374	10661	7866	9788	8848	11041
13	hCG2001263	CLEC4E	C-type lectin domain family 4, member E	12p13.31	Immunity and defense|Other immune and defense|Macrophage-mediated immunity;Immunity and defense	12187	17652	820	214	1631	2670	1237	1092	654
14	hCG22476.3	HSD11B1	hydroxysteroid (11-beta) dehydrogenase 1	1q32-q41	Other metabolism	9132	8073	2877	768	456	221	180	441	136
15	hCG1644254.2	null	null	null	null	21739	25632	4092	6795	7498	4936	5950	5682	10090
16	hCG1811376.3	SPOCK3	sparc/osteonectin, cwcv and kazal-like domains proteoglycan (testican) 3	4q32.3	Biological process unclassified	1152	907	209	293	108	246	281	218	262
17	hCG15754.3	ASB9	ankyrin repeat and SOCS box-containing 9	null	Biological process unclassified	8115	6341	1061	2079	2250	1631	1095	2330	1754
18	hCG16018.3	RARRES1	retinoic acid receptor responder (tazarotene induced) 1	3q25.32	null	6447	4418	1068	377	696	560	435	902	523
19	hCG25207.4	null	null	null	null	4037	3765	1932	1296	1901	2124	1886	1796	1646
20	hCG23356.2	HOXC5	homeobox C5	12q13.3	Nucleoside, nucleotide and nucleic acid metabolism|mRNA transcription regulation;Developmental	3043	2926	1466	1483	1691	1405	1669	1734	1885

					processes|Ectoderm development|Segment specification;Developmental processes|mRNA transcription|Neurogenesis									
21	hCG2040298	null	null	null	null	1528	1454	380	161	414	544	348	572	302
22	hCG1746984.1	IGFBP7	insulin-like growth factor binding protein 7	4q12	Cell proliferation and differentiation	79772	66820	25117	6618	6235	14063	10050	21058	6616
23	hCG1660428.2	KSP37	null	4p16	Biological process unclassified	3755	2426	163	154	110	451	215	376	81
24	hCG1818783.1	null	null	null	null	487	480	205	254	236	168	210	195	192
25	hCG2018311	LOC142937	null	10p15.1	null	1106	867	220	117	353	269	260	208	215
26	hCG27117.3	PTD015	null	11q13.5	null	15088	15544	8581	11052	8989	8994	9933	8774	9782
27	hCG1813420.3	GRHL3	grainyhead-like 3 (Drosophila)	1p36.11	mRNA transcription|mRNA transcription initiation|Nucleoside, nucleotide and nucleic acid metabolism	10077	10100	4558	1182	1353	1237	781	1649	1264
28	hCG1811538.1	MYCBP2	MYC binding protein 2	13q22	Proteolysis;Developmental processes|Ectoderm development|Protein metabolism and modification|Neurogenesis	6168	6496	4191	3987	4279	4611	4532	4308	4789
29	hCG1811682.1	SELPLG	selectin P ligand	12q24	Biological process unclassified	14109	14783	7402	6786	6637	7016	8230	7651	9592
30	hCG2011423	GLDC	glycine dehydrogenase (decarboxylating; glycine decarboxylase, glycine cleavage system protein P)	9p22	Amino acid catabolism|Amino acid metabolism	2225	2800	6782	7893	7251	6383	7380	8217	8387
31	hCG31853.2	MLC1	megalencephalic leukoencephalopathy with subcortical cysts 1	22q13.33	null	440	452	141	148	83	107	138	64	173
32	hCG28310.3	CDKN2B	cyclin-dependent kinase inhibitor 2B (p15, inhibits CDK4)	9p21	Tumor suppressor|Cell cycle|Cell cycle control;Oncogenesis	3354	3435	1151	598	180	1126	304	1064	904
33	hCG24118.4	P4HA2	procollagen-proline, 2-oxoglutarate 4-dioxygenase (proline 4-hydroxylase), alpha polypeptide II	5q31	Protein metabolism and modification|Protein modification	28515	27117	18849	17487	14891	13802	16607	17696	17181
34	hCG1808625.2	LOC392997|LOC442726	null	null	Protein metabolism and modification|Protein biosynthesis	603	605	171	159	247	299	257	226	237
35	hCG39980.2	FGD4	FYVE, RhoGEF and PH domain containing 4	12p11.21	Skeletal development;Cell structure and motility|Mesoderm development|Developmental processes	6504	5652	2448	2631	3008	2671	3124	2135	3511
36	hCG1650092.3	LOC149837	null	20p12.3	null	717	499	134	141	92	164	120	82	113
37	hCG1818640.1	null	null	null	null	4721	4313	2353	1457	1721	2180	1582	2504	1916
38	hCG1745900.1	C10orf54	chromosome 10 open reading frame 54	10q22.3	null	43456	38017	20503	15248	10877	17857	14866	21843	16901
39	hCG1799365.1	null	null	null	null	2840	2166	875	1038	691	629	924	1122	872
40	hCG25667.3	CXCL12	chemokine (C-X-C motif) ligand 12 (stromal cell-derived factor 1)	10q11.1	Biological process unclassified	4657	5783	1803	2569	1602	1728	2371	1700	1237
41	hCG27798.3	PRSS23	protease, serine, 23	11q14.1	null	5043	3851	2377	1792	1916	2114	2005	1945	2222
42	hCG31810.2	VTN	vitronectin (serum spreading factor, somatomedin B, complement S-protein)	17q11	Signal transduction|Cell adhesion-mediated signaling;Cell adhesion|Cell communication	1964	1422	452	598	562	505	314	636	547
43	hCG21659.3	BMP5	bone morphogenetic protein 5	6p12.1	Skeletal development|Mesoderm development|Developmental processes	191	1571	6280	6391	6327	7200	8999	6584	7287
44	hCG18617.3	RNH1	ribonuclease/angiogenin inhibitor 1	11p15.5	Biological process unclassified	81697	79320	112846	105590	108627	112286	103649	109182	100150
45	hCG41203.3	LOC388886	null	22q11.23	null	9797	11016	3433	4230	5451	3872	4984	3134	5578
46	hCG28316.3	TIMP4	TIMP metallopeptidase inhibitor 4	3p25	Proteolysis|Protein metabolism and modification	1369	1435	167	146	237	568	476	320	72
47	hCG1744579.2	PDGFC	platelet derived growth factor C	4q32	Signal transduction|Ligand-mediated signaling;Developmental	9134	8636	3206	2941	2740	2572	2951	5387	3132

					processes|Cell communication|Other developmental process;Cell proliferation and differentiation									
48	hCG2039675	APOBEC3F	apolipoprotein B mRNA editing enzyme, catalytic polypeptide-like 3F	22q13.1	Nucleoside, nucleotide and nucleic acid metabolism|Pre-mRNA processing	989	967	312	409	279	315	416	511	226
49	hCG2024860	C10orf54	chromosome 10 open reading frame 54	10q22.3	null	12047	14068	6424	5510	2532	4660	3707	5604	3766
50	hCG21231.2	FNDC4	fibronectin type III domain containing 4	2p23.3	Biological process unclassified	3195	2860	4961	5966	5354	5490	4934	5737	5165
51	hCG19782.2	ZIC3	Zic family member 3 heterotaxy 1 (odd-paired homolog, Drosophila)	Xq26.2	Nucleoside, nucleotide and nucleic acid metabolism|mRNA transcription regulation;Developmental processes|Ectoderm development|mRNA transcription|Neurogenesis|Embryogenesis;Developmental processes	3033	3426	635	1124	1267	1116	876	1257	1710
52	hCG32195.2	LHFP	lipoma HMGIC fusion partner	13q12	Biological process unclassified	19367	18639	8241	8705	11815	8504	9757	11826	10969
53	hCG1793614.2	null	null	null	null	1583	1310	561	188	231	483	548	251	419
54	hCG2018581	ZFP64	zinc finger protein 64 homolog (mouse)	20q13.2	Biological process unclassified	3512	3852	2848	2872	2727	2857	2602	2841	2898
55	hCG38329.2	H2AFY	H2A histone family, member Y	5q31.3-q32	Nucleoside, nucleotide and nucleic acid metabolism|Chromatin packaging and remodeling	42029	41905	31835	27437	23664	28174	28329	25355	25996
56	hCG40834.2	CD302	CD302 antigen	2q24.2	Endocytosis|Intracellular protein traffic	4201	3987	2539	1677	2063	2340	2549	2088	2401
57	hCG2006314.3	null	null	null	null	690	795	269	399	215	237	189	211	204
58	hCG19943.2	C3orf37	chromosome 3 open reading frame 37	3q21.3	Biological process unclassified	10572	9949	11996	11860	11801	12008	12031	12434	12482
59	hCG39069.2	null	null	null	null	75462	72302	57252	61400	59475	56359	54378	52624	55460
60	hCG23731.3	HSPC128	null	12q21.31	Biological process unclassified	15387	13731	9453	10489	10988	10368	10902	9513	10073
61	hCG2023783.1	null	null	null	null	798	1071	244	186	169	149	405	223	271
62	hCG1997566	LOC440786	null	22q11.1	Immunity and defense|B-cell- and antibody-mediated immunity	4108	4766	1671	1770	1898	2596	2393	1809	2448
63	hCG1815500.1	LOC400120	null	13q13.3	null	1254	1677	682	423	357	251	384	266	301
64	hCG37091.3	MYOC	myocilin, trabecular meshwork inducible glucocorticoid response	1q23-q24	Vision;Cell structure and motility|Signal transduction|Cell surface receptor mediated signal transduction;Sensory perception	7310	8029	588	1928	2916	613	1235	2176	3282
65	hCG27498.3	PPP1R12A	protein phosphatase 1, regulatory (inhibitor) subunit 12A	12q15-q21	Protein phosphorylation|Protein metabolism and modification|Protein modification	3002	4855	7158	8477	7872	8219	8318	8472	7416
66	hCG2042586	null	null	null	null	367	373	694	915	819	791	828	783	749
67	hCG2029377	MGC4659	null	14q32.33	null	338	304	717	951	877	756	762	820	793
68	hCG39914.4	SARS	seryl-tRNA synthetase	1p13.3-p13.1	Amino acid activation|Protein metabolism and modification	12495	16084	22640	20889	20791	21041	20913	21868	20310
69	hCG38301.2	null	null	null	null	473	543	182	160	148	227	211	271	177
70	hCG17980.4	SEMA3C	sema domain, immunoglobulin domain (Ig), short basic domain, secreted, (semaphorin) 3C	7q21-q31	Other receptor mediated signaling pathway;Developmental processes|Angiogenesis;Developmental processes|Ectoderm development|Cell surface receptor mediated signal transduction|Signal transduction|Mesoderm development|Receptor protein tyrosine kinase signaling pathway;Signal transduction|Neurogenesis;Developmental processes|Heart development	40142	76418	178488	210951	258182	240260	271049	261862	246059
71	hCG18974.3	CNKSR2	connector enhancer of kinase	Xp22.12	Biological process unclassified	912	1253	336	457	446	466	449	356	300

			suppressor of Ras 2											
72	hCG27883.2	MCF2L	MCF.2 cell line derived transforming sequence-like	13q34	Oncogene;Cell structure and motility|Cell motility|Signal transduction|Intracellular signaling cascade;Oncogenesis	8097	6513	2532	2342	3438	3689	2153	3259	3836
73	hCG15406.3	null	null	null	null	3174	6148	185	277	201	324	172	231	282
74	hCG15384.3	KIF25	kinesin family member 25	6q27	Cell structure|Mitosis;Cell proliferation and differentiation;Cell structure and motility|Intracellular protein traffic;Protein targeting and localization;Developmental processes;Cell cycle	1040	749	178	278	246	385	173	315	206
75	hCG41127.3	FAM13C1	family with sequence similarity 13, member C1	10q21.1	Biological process unclassified	668	780	336	229	268	355	177	208	156
76	hCG31497.3	AGL	amylo-1, 6-glucosidase, 4-alpha-glucanotransferase (glycogen debranching enzyme, glycogen storage disease type III)	1p21	Biological process unclassified	22993	27417	9299	13836	16031	12598	10691	12799	12877
77	hCG2039233	FLJ32252	null	16p13.3	null	7459	6552	3195	3941	4777	3351	2904	3663	3847
78	hCG43689.4	LST1	leukocyte specific transcript 1	6p21.3	null	5577	5458	3161	2400	2937	3262	3064	2164	3655
79	R26_hCT1814004	null	null	null	null	608	672	320	168	350	248	204	221	205
80	hCG1644309.4	null	null	null	null	74297	67866	98389	91856	85228	96554	93683	94446	93851
81	hCG22929.3	KLK3	kallikrein 3, (prostate specific antigen)	19q13.41	Proteolysis|Protein metabolism and modification	398	487	160	154	144	104	134	179	229
82	hCG1998300	IQCF3	IQ motif containing F3	3p21.2	Biological process unclassified	623	452	127	205	143	185	104	220	126
83	hCG16924.2	TDRKH	tudor and KH domain containing	1q21	Nucleoside, nucleotide and nucleic acid metabolism	881	687	1513	1492	1512	1313	1312	1413	1620
84	hCG15506.3	KIAA1033	KIAA1033	12q24.11	null	15990	17431	11645	12156	11957	13908	11509	12148	12631
85	hCG41362.2	TMPIT	null	7q11.23	Biological process unclassified	3055	2341	1493	1289	1604	1529	1275	1344	1584
86	hCG16683.4	CCND3	cyclin D3	6p21	Mitosis;Cell proliferation and differentiation|Cell cycle control;Cell cycle|Cell cycle	8467	7625	4036	3308	3335	3871	5650	3971	3497
87	hCG2007960	SLC6A8	solute carrier family 6 (neurotransmitter transporter, creatine), member 8	Xq28	Transport|Small molecule transport;Transport|Extracellular transport and import	5913	6135	9390	11643	11253	10877	10312	10313	9223
88	hCG37576.2	IL7R	interleukin 7 receptor	5p13	null	5929	9271	859	496	1306	2733	605	2243	612
89	hCG2041919	null	null	null	null	848	985	217	337	422	254	193	479	256
90	hCG2012339	null	null	null	null	600	513	166	178	121	161	165	84	295
91	hCG40703.2	PROM1	prominin 1	4p15.32	Intracellular protein traffic|Other intracellular protein traffic	450	443	226	207	237	185	157	116	205
92	hCG2004016.1	LOC348094	null	15q22.31	Biological process unclassified	474	430	271	228	271	188	206	204	239
93	hCG17533.3	HSPC135	null	3q13.2	Protein metabolism and modification|Protein biosynthesis	4198	4438	3827	3511	3581	3686	3703	3551	3537
94	hCG16241.2	NCALD	neurocalcin delta	8q22-q23	Vision|Sensory perception	3161	1493	11427	15979	10087	13953	13010	11327	10811
95	hCG27553.2	IL1R2	interleukin 1 receptor, type II	2q12-q22	Cell surface receptor mediated signal transduction|Signal transduction|Cytokine and chemokine mediated signaling pathway	2067	2547	575	152	86	330	1151	273	162
96	hCG2002502	UNQ501	null	19p13.2	Biological process unclassified	59540	61000	113613	123006	111891	111891	107261	89410	104427
97	hCG43768.2	IGFBP6	insulin-like growth factor binding protein 6	12q13	Signal transduction|Growth factor homeostasis|Extracellular matrix protein-mediated signaling;Homeostasis|Cell communication	381756	253747	117909	96912	46098	53707	61056	134002	66706
98	hCG20563.2	FSTL3	follistatin-like 3 (secreted glycoprotein)	19p13	Homeostasis	12612	8471	5097	4706	3936	3271	3438	3213	3031

99	hCG1791193.1	null	null	null	null	45419	47071	27466	32102	28819	36104	28854	30722	25240
100	hCG25177.3	DDB2/LHX3	damage-specific DNA binding protein 2, 48kDa|LIM homeobox 3	11p12	p11 Nucleoside, nucleotide and nucleic acid metabolism|mRNA transcription regulation;Apoptosis|Biological process unclassified|Inhibition of apoptosis;Developmental processes|Ectoderm development|mRNA transcription|Neurogenesis;Oncogenesis|Embryogenesis;Developmental processes	3387	3465	1606	866	893	1512	901	1729	1888
